# Biocompatible Polymer/Quantum Dots Hybrid Materials: Current Status and Future Developments

**DOI:** 10.3390/jfb2040355

**Published:** 2011-12-02

**Authors:** Lei Shen

**Affiliations:** Department of Chemistry & Biochemistry, The University of Texas at Austin, TX 78712, USA; E-Mail: shenl@mail.utexas.edu; Tel.: +1-512-471-2589

**Keywords:** polymer, quantum dots (QDs), biocompatible

## Abstract

Quantum dots (QDs) are nanometer-sized semiconductor particles with tunable fluorescent optical property that can be adjusted by their chemical composition, size, or shape. In the past 10 years, they have been demonstrated as a powerful fluorescence tool for biological and biomedical applications, such as diagnostics, biosensing and biolabeling. QDs with high fluorescence quantum yield and optical stability are usually synthesized in organic solvents. In aqueous solution, however, their metallic toxicity, non-dissolubility and photo-luminescence instability prevent the direct utility of QDs in biological media. Polymers are widely used to cover and coat QDs for fabricating biocompatible QDs. Such hybrid materials can provide solubility and robust colloidal and optical stability in water. At the same time, polymers can carry ionic or reactive functional groups for incorporation into the end-use application of QDs, such as receptor targeting and cell attachment. This review provides an overview of the recent development of methods for generating biocompatible polymer/QDs hybrid materials with desirable properties. Polymers with different architectures, such as homo- and co-polymer, hyperbranched polymer, and polymeric nanogel, have been used to anchor and protect QDs. The resulted biocompatible polymer/QDs hybrid materials show successful applications in the fields of bioimaging and biosensing. While considerable progress has been made in the design of biocompatible polymer/QDs materials, the research challenges and future developments in this area should affect the technologies of biomaterials and biosensors and result in even better biocompatible polymer/QDs hybrid materials.

## 1. Introduction

In the life sciences, fluorescence is widely used as a significant technique for people to study and understand the biological structure of organism, the cell-cell interaction and the interplay of biomolecules. In this technique, kinds of fluorophores are developed to label, detect and image the bio-targets. These fluorophores are small molecules, proteins or quantum dots (QDs).

QDs are semiconductor nanoparticles with the three dimensions confined to 2–10 nm length scale [1]. They are usually composed of groups II-VI or III-V atoms in the periodic table. The fluorescence of small molecules contributes to delocalized electrons which can jump a band and stabilize the energy absorbed, while QDs, in a different way, show fluorescence by quantizing their semiconductor energy level smaller than their nanometer sized radius. Compared with small fluorescent molecules and protein fluorophores, QDs have attracted more tremendous attention to biologists and chemists principally because of three main reasons [2,3]. First, the wavelength of the band-edge adsorption and fluorescence emission of QDs can be tuned systematically by changing their size. Second, the photoluminescence spectra of QDs can be detected in a wide wavelength region, from visible spectrum to near-infared, by a single excitation source. Third, QDs have long luminescent life and are extremely photostable, and therefore they can be used for dynamic imaging of living cells.

**Figure 1:**
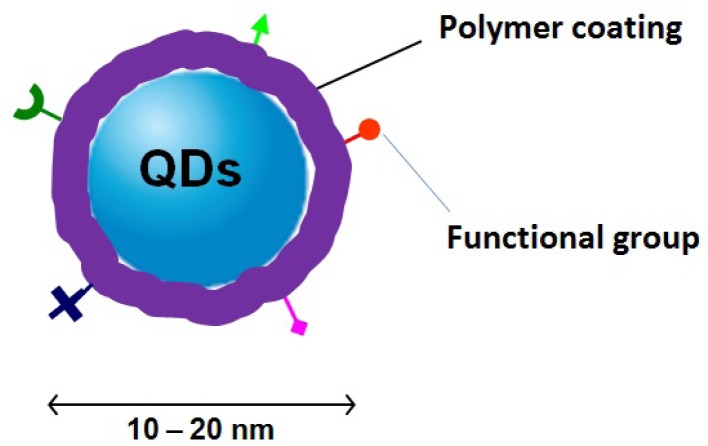
A schematic depiction of polymer/QDs hybrids. Biocompatible polymers protect QDs as shells providing biocompatibility and biostability and, at the same time, introduce functional groups for targeting cell and biomolecules.

High photoluminescence quality QDs are usually synthesized in organic solvents through high-temperature routes. The as-grown QDs are normally covered by small hydrophobic molecules (e.g., triocylphosphine oxide or hexadecylamine) so that they have no intrinsic aqueous solubility, which limits their biological applications. Another limitation is that the physical stability of QDs is easily disrupted through simple processing steps in water. When transferred into water, QDs tend to aggregate, which decreases the fluorescence quantum yields of QDs. Moreover, QDs are made from toxic elements against aqueous organism. Because of their non-dissolubility, photoluminescence instability and metallic toxicity in water, QDs are usually required to be modulated by passivation process, whereby other hydrophilic coating materials bind or coordinate to QDs surface, to provide biocompatibility and bio-stability. To achieve this goal, polymers with excellent biocompatibility and low toxicity are successfully and widely employed to modify QDs surface and engineer biocompatible QDs composites for a variety of medical and biological applications [4,5]. As the structure presented in Figure 1, polymers provide surface passivation for QDs and protect them as a stable interface between QDs and biological networks. At the same time, polymers decrease the toxicity of QDs. In addition, polymers can introduce functional groups for QDs to fulfill their end-use application, such as receptor targeting and cell attachment.

Although the mixing of polymer and nanoparticles is not a novel scientific project [6], the development of better polymer/QDs materials that exhibit advantageous biocompatible and optical properties has nowadays been an emerging research field. Polymers with different architectures, such as homo- and co-polymer, hyperbranched polymer, and polymeric nanogel, have been employed to anchor and coat QDs for fabricating biocompatible polymer/QDs hybrid materials. This current review summarizes the recent development of methods for the preparation of these hybrid materials and describes their applications in the fields of bioimaging and biosensing. The research challenges and future developments in the area of biocompatible polymer/QDs hybrid materials are also presented.

## 2. How to Make Biocompatible Polymer/QDs Hybrids?

**Figure 2:**
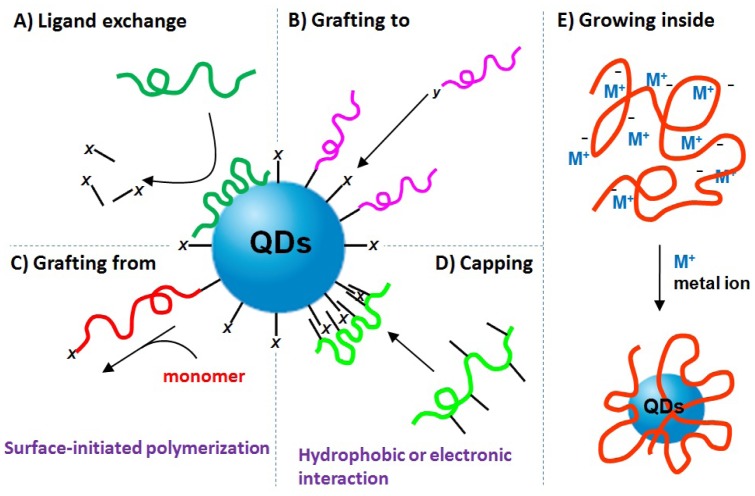
Schematic summary of synthetic strategies for fabricating biocompatible polymer/QDs hybrid materials, which can be categorized into (**A**) ligand exchange between polymer and QDs; (**B**) grafting polymer to QDs; (**C**) grafting polymer from QDs; (**D**) capping polymer onto QDs; and (**E**) growing QDs within polymeric template.

**Figure 3:**
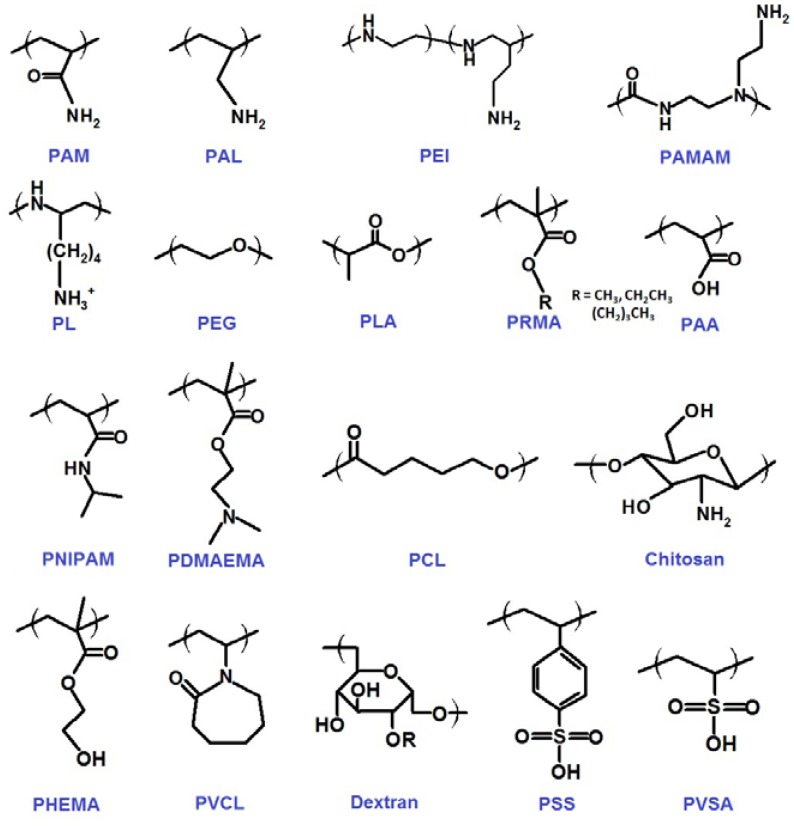
Chemical structures of biocompatible polymers employed for the fabrication of polymer/QDs hybrid materials described in this review.

As described above, QDs are normally synthesized by an organometallic route involving high temperature and consist of a metallic core surrounded by a shell of capping ligands. These ligands, with polar head groups and hydrophobic organic tails, make QDs insoluble in water. The incorporation of QDs into biological systems often requires strategies for the manipulation of the ligands bound to the QDs surface to make them water-soluble and biocompatible. In the past 10 years, five major synthetic strategies have been developed to generate biocompatible polymer/QDs hybrid materials as provided in Figure 2, which include: (A) ligand exchange between polymer and QDs; (B) grafting polymer to QDs; (C) grafting polymer from QDs; (D) capping polymer onto QDs; (E) growing QDs within polymer template. A variety of biocompatible polymers (Figure 3) have been employed to modify QDs surface.

### 2.1. Ligand Exchange Between Polymer and QDs

Ligand exchange has been extensively used by many research groups as an approach to modify the surface of colloidal QDs. The ligand exchange procedure entails replacing the as-grown ligand (e.g., phosphine oxide) introduced during the QDs synthesis with new biocompatible polymers (Figure 2(A)). These biocompatible polymers usually have functional anchor groups, such as thiol, amine and carboxyl, which can passivate QDs more strongly than the original ligand.

Biocompatible polymers with thiols have been proven useful in ligand exchange due to thiol's high affinity for metallic surface. In a typical example, Uyeda *et al.* [7] and Yildiz *et al.* [8] employed the thiol terminated poly(ethylene glycol) (PEG), which is a well-known biocompatible polymer with many applications from industrial manufacturing to medicine, to prepare biocompatible QDs fluorophores. Recently, Wu *et al.* [9] synthesized thiol-PEG-peptide hybrid polymers and used them for preparingpH-responsive QDs. The resulted polymer/QDs show great dispersity and biocompatibility in water. Besides PEG, thiol terminated OH-poly(amidoamine) (OH-PAMAM) hyperbranched polymers was exploited for stabilizing QDs in aqueous systems [10]. Nevertheless, thiol-stabilized QDs are found to be unstable due to photooxidation of thiols and reduced photoluminescence of as-grown QDs.

**Figure 4:**
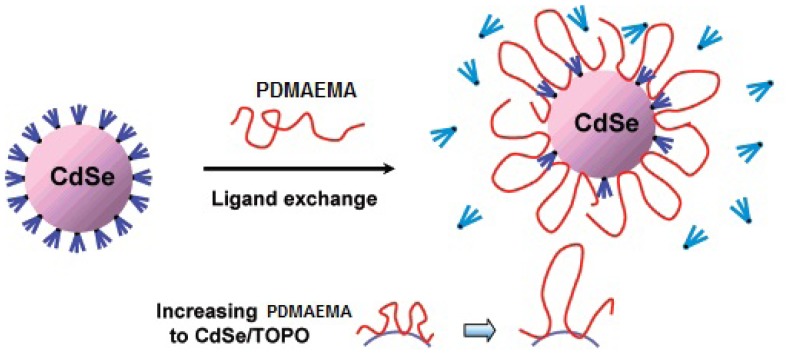
A schematic representation of ligand exchange process of poly(2-(dimethy-lamino)ethyl methacrylate) (PDMAEMA) with CdSe QDs, where the polymer binds to QDs in the form of small loops. 3% of PDMAEMA chain interacts directly with QDs as train structures and 97% is present in form of loops and tails protruding into solution. Reprinted with permission from reference [17].

Other research groups have demonstrated the utility of amine functionalized polymers as ligands for ligand exchange process. Amine –NH_2_ modified poly(acrylic acid) (PAA), an innoxious and water-soluble polymer, has been used to generate PAA/QDs hybrids with long-term colloidal stability [11,12]. –NH_2_ functionalized poly(acryloyoxysuccinimide) (PAAS) was also introduced to modify QDs surface [13]. Moreover, the transfer of QDs into aqueous solution using –NH_2_ modified PEG by ligand exchange method was also presented [14]. Recently, pendent tertiary amine group has been demonstrated to have the intendancy to efficiently replace the original ligands of QDs and anchor QDs. For instance, Wang *et al.* [15,16] reported the efficient utility of poly(2-(dimethy-lamino)ethyl methacrylate) (PDMAEMA) (PDMAEMA), which is a thermo- and pH sensitive biocompatible polymer with tertiary amines, to modify QDs through ligand exchange. The resulted PDMAEMA/QDs can provide robust colloidal stability and enhance the brightness of QDs. Furthermore, in order to find out the relationship between the physical structures of polymer on QDs and the quantum yields of polymer/QDs hybrids, Shen *et al.* studied the interaction of PDMAEMA with QDs and quantitatively examined the polymer-ligand exchange process [17]. They determined that 3% of PDMAEMA chain interacted directly with QDs as train structure and 97% was present in form of loops and tails protruding into solution (Figure 4).

Other kinds of amine functionalized polymers have also been employed to modify QDs surface through ligand exchange process, such as imidazole modified linear PEG [18], linear poly(allylamine) (PAL) [19], hyperbranched poly(ethyleneimine) (PEI) [20,21] and PAMAM [22], and imidazole functionalized poly(*N*-isopropylacrylamide) (PNIPAM) and poly(*N*-vinylcaprolactam) (PVCL) nanogels [23]. In Addition, some natural polymer, *i.e.*, chitosan a hydrophilic, biodegradable, biocompatible and nontoxic polymer with –NH_2_ group, has also been exploited to modify QDs by ligand exchange method for generating biocompatible polymer/QDs [24].

Besides amine and thiol, biocompatible polymers with other functional anchor groups have been synthesized and introduced onto QDs by ligand exchange procedure. For example, Bawendi *et al.* [25] synthesized PEG polymers with multidentate phosphine oxide ligand, which binds to QDs more efficiently than single phosphine oxide ligand, and generated biocompatible PEG/QDs. This hybrid material provided chemically stable and highly fluorescent properties. Moreover, carboxyl modified polymer, *i.e.*, PAA [26,27], can be used to displace original carboxyl ligands on PbS QDs and enables water-soluble and biocompatible properties. The above works show that ligand exchange provides an efficient method for researchers to fabricate biocompatible polymer/QDs hybrid materials.

### 2.2. Grafting Polymer to QDs

In “grafting to” method, functional polymers independently synthesized are covalently linked to QDs surface through the anchor groups at the end of or along the polymer chain (Figure 2(B)). Different linear and hyperbranched polymers have been “grafted to” QDs to generate biocompatible polymer/QDs hybrids.

In a typical example, linear PEG has been coupled with CdSe QDs through 1-ethyl-3-(3-dimethyl-aminopropyl) carbodiimide/N-hydroxysuccinimide (EDC/NHS) reaction [28,29]. Peng and coworkers have grafted hyberbranched PEI to CdSe QDs to generate biocompatible PEI/QDs with great water-solubility [30]. The resulted PEI/QDs have been demonstrated with thermal stability in a large temperature range needed.

Alternatively, “Grafting to” is a useful technique to couple biopolymers (e.g., protein, peptide and DNA) onto QDs. Since biopolymers have a number of –NH_2_ groups along the molecule chains, they can easily react with –COOH functionalized QDs through EDC/NHS chemistry. These biopolymer/QDs conjugates provide nice biocompatibility. For example, antibody proteins have been covalently “grafted to” highly luminescent CdSe/ZnS core/shell QDs for biological detection [31,32].

### 2.3. Grafting Polymer from QDs

Another successful method employed to generate biocompatible polymer/QDs is the “grafting from” chemistry, in which the polymer is grown outward from the QDs surface by surface-initiated polymerization chemistry (Figure 2(C)). The QDs are originally decorated with small molecule ligands from which the polymerization can be initiated. The polymerization techniques contain ring-opening polymerization (ROP) [33], reversible addition-fragmentation chain transfer (RAFT) polymerization [34], nitroxide-mediated radical polymerization (NMRP) [35], atom transfer radical polymerization (ATRP) [36,37] and oxyanionic vinyl polymerization (OAVP) [38].

In a typical example, poly(methy methacrylate) (PMMA), as a main biocompatible material used in a variety of medical applications from contact lenses to bone cements, was polymerized onto QDs by Patten's group [36] using ATRP and by Emrick's group [34] using RAFT. PMMA was also copolymerized with polystyrene onto QDs by Emrick's group using both RAFT [34] and NMRP [35]. With a similar structure as PMMA, poly(butyl acrylate) was successfully exploited to modify QDs by Barros-Timmons' group [37] using ATRP and by Emrick's group [34] using RAFT.

Carrot *et al.* [33] employed ROP to polymerize poly(caprolactone) (PCL), a biocompatible and biodegradable polymer, onto QDs. With the easy degradability of PCL by hydrolysis of its ester linkages in physiological conditions (such as in the human body), PCL/QDs therefore may receive a great deal of attention for use as an implantable biomaterial and also as drug release and delivery vehicle. Besides PCL, hyperbranched PEG was also “grafted from” QDs through ROP to fabricate biocompatible PEG/QDs material [39] Additionally, Zhou *et al.* [38] reported the OAVP method to generate PDMAEMA/QDs material. This biocompatible hybrid material has been demonstrated with excellent aqueous solubility and stable photoluminescence.

### 2.4. Capping Polymer onto QDs

The fourth widely used strategy to fabricate polymer/QDs hybrids is to cap polymers onto QDs surface through physical interaction, such as hydrophobic or electronic interaction, between polymers and original ligands on QDs (Figure 2(D)). A number of amphiphilic copolymers and polyelectrolytes were synthesized to modify QDs surface through capping method.

Amphiphilic copolymers couple the original ligands of QDs with hydrophobic segments and expose hydrophilic parts into water to promote QDs dispersion in aqueous media. In a typical example, extensive works have demonstrated that amphiphilic PEG polymers can well encapsulate QDs as the hydrophobic core and stabilize QDs in water [40,41,42,43]. The resulted biocompatible PEG/QDs have been shown to have the potential to detect and image biomolecules. In addition, alkyl-modified amphiphilic PAA have been employed to coat QDs through capping method for bio-detection [44,45,46,47]. Because of easy hydrolysis into PAA, poly(maleic anhydride) copolymers have also been widely used to make QDs with great biocompatibility through capping method [48,49,50,51,52,53].

On the other hand, polyelectrolytes can “cap onto” QDs surface by electrostatic interaction. For example, a multifunctional poly(acrylamide) (PAM) was synthesized to modify CdSe/ZnS core/shell QDs through electrostatic interaction between the positively charged side chains of PAM and the negatively charged ligands on QDs [54] As a special kind of polyelectrolytes, biopolymers can also couple with QDs through capping method by electrostatic interaction for generating biocompatible conjugates. For instance, positively charged DNA molecules were conjugated with negatively charged CdSe/ZnS QDs for luminescence and bioassays applications [55].

**Figure 5:**
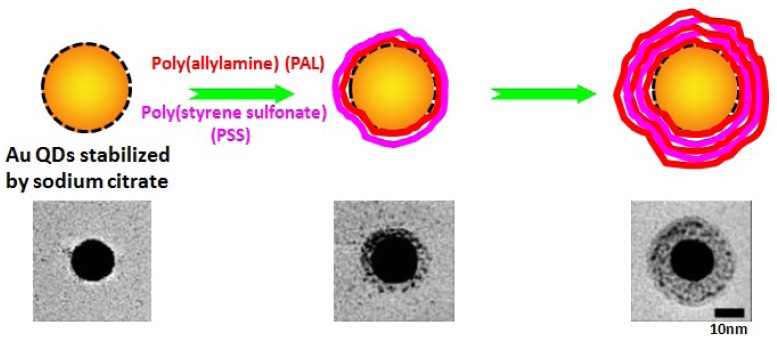
Single PSS/PAL/Gold nanoparticle through layer-by-layer capping method. Below are transmission electron micrographs of hybrid nanoparticles with different number of PSS/PAL layers. Reprinted with permission from reference [56].

Compared with “grafting from” and “grafting to”, capping method can generate more compact polymer/QDs hybrids with minimized hydrodynamic sizes in water because of the multi-interaction between QDs and single polymer chain. In order to generate different kinds of polymer/QDs conjugates with the same QDs core, researchers have employed different polyelectrolytes with opposite charges to sequentially coat QDs through layer-by-layer assembly. In a typical example, up to 20 layers of polyanion poly(styrene sulfonate) (PSS) and polycation PAL have been used to consecutively coat single gold QDs (Figure 5) [56]. The resulted biocompatible PSS/PAL/QDs were well colloidal stable in water. Other polyelectrolyte pairs, e.g., positive polylysine (PL)/negative dextran [57] and positive PAL/negative poly(vinylsulfonic acid) (PVSA) [58], have also been subsequently deposited onto QDs to generate biocompatible polymer/QDs hybrids. The layer-by-layer capping method generates nice colloidal stable polymer/QDs nanoparticles and opens a new route for functionalizing QDs with desirable properties for biological application.

### 2.5. Growth of QDs Within Polymer

The last route to prepare biocompatible polymer/QDs utilizes polymers as the template to directly synthesize QDs inside (Figure 2(E)). The biocompatible polymers used in this method can be linear, hyperbranched or nanospherical polymers with the metal ion-containing segments. The metal ions are then chemically transformed via reduction or precipitation reactions into QDs nanoparticles. Through this method, QDs can be grown inside polymer and polymer acts as the protective shell for QDs in aqueous solution. For example, PAMAM dendrimer [59] and PMMA polymeric microsphere [60,61] were used to fabricate highly luminescent PAMAM/CdS and PMMA/CdS QDs hybrid materials. Zhang *et al.* [62] reported the employment of PNIPAM nanogel for kinds of QDs synthesis inside, such as semiconductor, metal and magnetic nanoparticles. The generated PNIPAM/QDs retains both stimulus-responsive behavior of polymer and the optical property of QDs. Recently, Zhou *et al.* [63] have used hyperbranched PEG as a stabilizer to generally synthesize a variety of QDs (e.g., monometallic, alloy of noble metal, semiconductor, magnetic, rare-earth, and silver and gold nanocrystals) inside. By controlling the concentration of metal ions in solution and the metal ion-containing segments along polymers, we can adjust the sizes and the densities of QDs inside polymers.

## 3. Biocompatible Polymer/QDs for Biolabeling and Bioimaging

Since the independently reports of Alivisato's [64] and Nie's groups [31] on the ability of QDs to label biomolecules in 1998, significant progress has been made in the field of QDs for biological labeling and imaging [65]. The resistance to photo-bleaching, narrow emission, high energy absorption and single source excitation all are strong reasons to make QDs superior fluorescence labeling agents as opposed to small molecule dyes. Because QDs are generally made by toxic metal elements and insoluble in water, researchers move their efforts forward to biocompatible QDs fabrication. With better biocompatibility and lower toxicity, polymers is one, somehow the most part, of the best coating meterials that can render the QDs water soluble and at the same time equip them with functional groups for labeling and imaging bio-tags.

**Figure 6:**
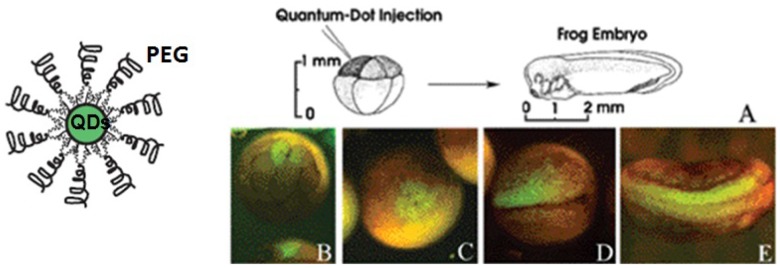
PEG/CdSe QDs labeling of Xenopus embryos at different stages. (**A**) Schematic showing the experimental strategy; (**B**) Injection of one cell out of an eight-cell-stage embryo resulted in labeling of individual blastomeres; (**C**) Same embryo shown 1 h later; The daughter cells of the injected blastomere are labeled (**D**) and at a later stage (**E**) show two neurula embryos, which were injected into single cell at the eight-cell-stage in the animal pole. Reproduced with permission from ref 40.

For labeling and imaging bio-tags, biocompatible polymer/QDs hybrids should satisfy the following criteria: (1) the hybrid particles should keep stable under most biological environments and non-specific to biological cellular systems while still permitting bioconjugation; (2) the thickness of polymer shell for modifying QDs surface should be appropriate to promote for easy uptake of these particles by cells, *i.e.*, the kinetics of nonspecific endocytosis by cells are highly dependent on the size of hybrid particles [66]. The smaller the QDs, the more efficient the endocytosis process. Numerous reports have been published describing the use of polymer/QDs to label and image cells. For example, PEG is an ideal polymer because it can reduce nonspecific adsorption of biomolecules on QDs. Dubertret *et al.* [40] prepared PEG/CdSe hybrid nanoparticles and demonstrated their *in vivo* imaging (Figure 6). After injected into and labeled with Xenopus embryos, the PEG/CdSe were stable, nontoxic, cell autonomous, and slow to photobleach. Other groups also independently used PEG/QDs for bioimaging. For instance, Zhou *et al.* [39] employed hyperbranched PEG/QDs to label and image human lung cancer cells SPCAI without fluorescence quenching within at least 24 h. Ballou *et al.* [28] demonstrated the long-term fluorescence of PEG/QDs for four months in mice for imaging and detection. In addition, Nie and coworkers [21] systhesized PEG-grafted-PEI hyperbranched ligands to modify QDs. Compared with previously reported polymer/QDs, the resulted PEG/QDs were smaller in size and more stable in acidic environments. These PEG/QDs were rapidly internalized by endocytosis for intracellular imaging and therapeutic applications. The above examples demonstrate the successful application of PEG/QDs hybrids for bio-labeling and bioimaging applications.

**Figure 7:**
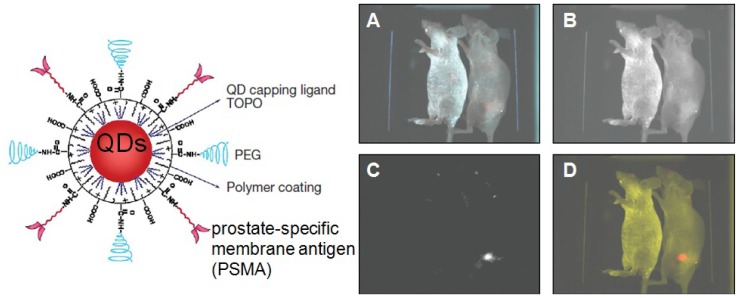
Spectral imaging of QD-PSMA Ab conjugates in live animals harboring C4-2 tumor xenografts. Orange-red fluorescence signals indicate a prostate tumor growing in a live mouse (right). Control studies using a healthy mouse (no tumor) and the same amount of QD injection showed no localized fluorescence signals (left). (**A**) Original image; (**B**) unmixed autofluorescence image; (**C**) unmixed QD image; and (**D**) super-imposed image. After *in vivo* imaging, histological and immunocytochemical examinations confirmed that the QD signals came from an underlying tumor. Note that QDs in deep organs such as liver and spleen were not detected because of the limited penetration depth of visible light. Reproduced with permission from Reference [41].

Other biocompatible polymer/QDs systems were also exploited for biological labeling and imaging. Larson *et al.* [45] used PAA/CdSe nanoparticles as fluorescent labels for multiphoton microscopy which enables multicolor imaging in demanding biological environments such as living tissue. They visualized QDs dynamically through the skin of living mice and found no evidence of blinking of QDs in solution. Using the same PAA/QDs system, Wu *et al.* [44] labeled the breast cancer cells marker Her2 on the surface of live cancer cells to detect nuclear antigens inside the nucleus. By this method, they detected several cellular targets with one excitation wavelength. Additionally, Nie's group [41] designed an alkane-PEG-PAA triblock copolymer and linked this amphiphilic polymer to tumor-targeting ligands and drug-delivery functionalities (Figure 7). This system was demonstrated to efficiently target animal prostate cancer cell both by the enhanced permeability and retention of tumor sites and by antibody binding to cancer-specific cell surface biomarkers. The above results indicate that biocompatible PAA/QDs can be very effective in cellular imaging and multiplex bio-target labeling.

**Figure 8:**
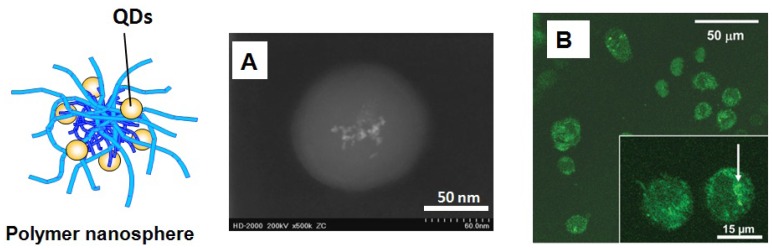
Polymer nanosphere/QDs hybrids. (**A**) Dark-field TEM image of one hybrid poly(N-vinylcaprolactam)/Lanthanide QDs (PVCL/La) particle. (**B**) Laser confocal fluorescence microscopy (LCFM) image of THP-1 cells labeled by PVCL/La. Reproduced with permission from Reference [67].

Besides the above works, various biocompatible polymeric nanospheres containing QDs have been incorporated into cell for detecting and imaging. Compared with thin polymer layer on single QD, polymeric nanospheres can provide more space for loading more QDs and carry more functional active groups for bioconjugation (Figure 8). In a typical example, Pich *et al.* [67] synthesized biocompatible PVCL/Lanthanide QDs hybrids with excellent colloidal and thermo-sensitive properties and these hybrid particles are easily taken up by human monocytic leukemia cell THP-1 (Figure 8). In this work, the elemental composition of QDs and the signal intensity indicated their type and number, respectively, and are used to barcode live cells. Pan *et al.* [68] and Guo *et al.* [69] used poly(lactide) (PLA) nanosphere containing CdSe QDs to target and image fibrioblast cell NIH 3T3 and breast cancer cell MCF-7. Such biodegradable PLA/CdSe provides better cytotoxicity for normal cell such as NIH 3T3 than MCF-7. Wu and coworkers [70] designed polysaccharide-based nanogels to immobilize CdSe inside for tumor cell imaging. In addition, Lin *et al.* [71] reported the utility of water-soluble chitosan/CdSe/ZnS hybrid nanospheres for biological imaging and labeling both *in vitro* and *in vivo*. However, the bottleneck of polymer nanosphere/QDs system is the size issue of hybrid particle which should satisfy the aforesaid criteria for bioimaging and biolabeling. How to generate smaller polymeric particle for probe is still a challenge in the field of polymer nanospheres/QDs hybrids.

Recently biodiagnostics requires deep-tissue imaging with emission far red/near infrared wavelength. The tumor imaging sensitivity is optimized if the excitation and emission of polymer/QDs can occur at wavelengths where the major absorption peaks of blood and water are absent. Cai *et al.* [72] used arginine-glycine-aspartic acid peptide modified PEG/QDs for in vivo targeting and imaging of tumor vasculature. This result opens up new perspectives for integrin-targeted near-infrared optical imaging and cancer detection by using biocompatible polymer/QDs hybrid materials.

## 4. Biocompatible Polymer/QDs for Biosensors

Due to the aforementioned unique spectral properties and physicochemical stability compared with small molecular fluorophores, QDs have the potential for biosensing applications, such as immuneassays detection and fluorescence resonance energy transfer (FRET) based sensing [73]. The large surface area of polymer coating on QDs enables conjugation of multiple copies of various biomolecules, such as proteins, peptides and DNA. This property renders QDs an attractive nano-scafold for multifunctional immunoassays detection. For example, poly(maleic anhydride) (PMA) copolymers was employed by Sanz-Medel's group [49] for the bioconjugation of QDs with antibodies. PMA can easily hydrolyze into PAA and then react with antibody via reaction with ethyl-3-(dimethyl-aminopropyl) carbodiimide (EDC). This simple bioconjugation of polymer/QDs with antibodies was demonstrated for fluorescence-based immunoassays to detect aflatoxin B1 (AFB1) molecule, which is toxic and carcinogenic to humans and animals and frequently contaminates agricultural commodities.

**Figure 9:**
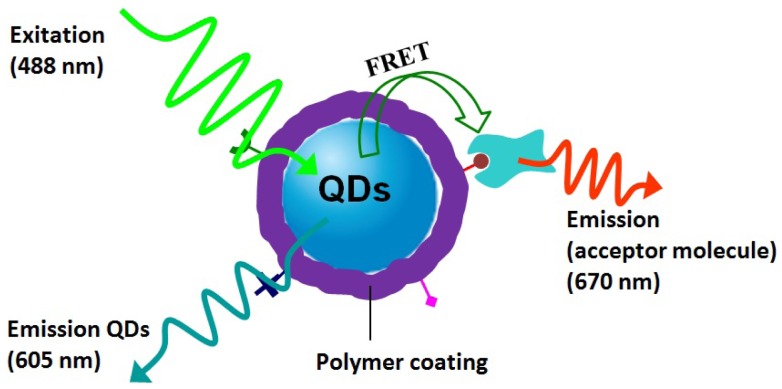
A schematic structure of a single-polymer/QDs-based biosensor in the presence of acceptor molecule with fluorescence emission at 670 nm and illumination on QDs cased by fluorescence resonance energy transfer (FRET) between acceptor molecule and QDs donor. Polymer introduces functional groups for targeting the acceptor molecules.

On the other hand, QDs have a longer excited-state lifetime compared to that of organic dyes, thus they have been widely used as donor part in FRET-based sensing assays. The idea here is to detect the target biomolecules through the fluorescence emission of the QD at required wavelength. Polymers have been widely exploited as the bridge between QDs donor and acceptor molecules in FRET sensing application (Figure 9). In a typical example, Potapova *et al.* [13] used poly(acryloyoxysuccinimide) (PAAS) as an versatile ligand for QDs modification. Decoration of the resulted PAAS/QDs with acceptor dye molecules allowed investigating FRET between QDs and the attached chromophores. This work showed that QDs-polymer-dye composites had higher photo-stability than QDs-dye systems which allowed FRET experiments on single composites to be performed. In addition, Duan *et al.* [50] developed multicolor PMA/CdSe/ZnS probes with compact sizes, robust colloidal stability and high quantum yields. The size-minimized polymer/QDs allowed fabrication of bioconjugated QDs by chelating between polyhistidine tags of recombinant proteins and QDs surface. The linked proteins were able to give highly efficient FRET between QDs and protein acceptor. Moreover, Diaz *et al.* [51] used the same polymer/QDs system to generate biocompatible, water-soluble and photoswitchable QDs. PMA/QDs has been demonstrated for successful fluorescent immunoassays. Recently, Bawendi's group [18] synthesized PEG/QDs hybrids with compact size (10–12 nm) and high quantum yields. By incorporating 5-carboxy-X-rhodamine (ROX), a red-emitting fluorescent dye, with PEG/QDs, they generated a highly efficient QD-polymer-dye energy transfer pair covalently conjugated with streptavidin for high-affinity single molecule imaging of biotinylated receptors on live cells. In short, the application of biocompatible polymer/QDs for FRET biosensor has been fully demonstrated.

It should be noted that highly efficient FRET requires the short distance between QDs donor and acceptor molecules on the 1–10 nm range. The disadvantage of polymer layers here is that they inevitably increase the distance between donor and acceptor and lower the FRET efficiency. Thus, in order to obtain highly ERET efficiency, the polymer shell should be made as thin as several nanometers range and carry more functional groups for conjugating enough acceptor molecules. How to design size-minimized polymer/QDs hybrids with compact polymer shell is the key step for their successful biosensing application.

## 5. Outlook and Future Challenges

Biocompatible polymer/QDs hybrid materials have been shown to have great potential in the fields of biological and medicine application. Polymer shell plays as a crucial role for tuning surface properties, imparting colloidal stability and incorporating functionality to QDs core for their end-use applications. Chemists have made marvelous accomplishments for generating different kinds of biocompatible polymer/QDs systems based on corresponding applications. Despite these advances, challenges remain for biocompatible polymer/QDs when adopted in biological applications and the field of biocompatible polymer/QDs probes and sensors can be further developed and exploited as described below.

First, interfaces and surfaces play important roles in determining the physical attributes of polymer/QDs hybrids. During polymer/QDs fabrication processes, polymers interact with and partially change the original QDs surface, which introduces “defect” and somehow decreases the highly quantum yields of as-synthesized QDs. Thus, there is an urgent need to develop polymers, control the manufacturing process and decrease “defect” sites on QDs so as to provide highly luminescent ultrabright polymer/QDs probes. Fundamentally understanding of polymer interaction with metallic QDs surface absolutely remains an important issue in polymer/QDs field and guides people to synthesize biocompatible polymer/QDs probes with ultrahigh luminescence.

Second, the size of polymer/QDs probe should be small enough for easy endocytosis by cells. Generally, the hydrodynamic sizes of polymer/QDs increase by 20–50 nm compared with that of as-grown QDs. In order to obtain highly efficient endocytosis, polymer/QDs should minimize their diameters but also keep the colloidal stability. Nie and coworkers [12] synthesized a multifunctional multidentate polymer ligand based on PAA to modify QDs surface. The resulted coating polymer layer is only 1.5–2.0 nm in thickness. This compact shell provides QDs of high brightness and stability in water. On the other hand, the size-minimized polymer/QDs hybrids open new possibility for biomolecular and cellular tracking at single molecule level [74]. Thus, how to generate minimized polymer/QDs probes for different bioapplications is the second coming challenge. A variety of well-designed polymers will be indispensable in minimizing polymer/QDs probes.

Third, not only in test-tube assays, the polymer/QDs should keep colloidal stability over a broad range of *in vivo* biological conditions, such as highly concentrated electrolyte solutions, different temperature windows, and wide range of pH values. Specifically, the polymer layers should prevent nonspecific protein adsorption on QDs and self-aggregation of QDs, which influence the luminescent efficiency of polymer/QDs probe. This requires researchers to generally develop more multifunctional polymer/QDs systems to facilitate their long-term, aggregation-free use in aqueous enviroment.

Fourth, polymer layers should be stable enough to prevent the possible release of toxic metal from QDs after injection *in vivo* [75]. The cytotoxicity of polymer/QDs in body has been poorly investigated and understood [29,76]. Development of new functional polymer coatings with low cytotoxicity is definitely needed for the bioapplications of polymer/QDs hybrid mateials.

Finally, although polymer layers provide stability and decrease the toxicity of QDs, we do not want polymer/QDs to stay in the body for long time after being detected and imaged. How to get polymer/QDs probe out of body remains a significant challenge for applications of these materials. Although polymer/QDs have gained successful and widely use *in vitro*, understanding how they move through the body will provide a breakthrough in this field and establish the guidelines for *in vivo* bioapplication of polymer/QDs hybrid materials.
